# Electron-beam-promoted fullerene dimerization in nanotubes: insights from DFT computations

**DOI:** 10.3762/bjoc.20.10

**Published:** 2024-01-17

**Authors:** Laura Abella, Gerard Novell-Leruth, Josep M Ricart, Josep M Poblet, Antonio Rodríguez-Fortea

**Affiliations:** 1 Departament de Química Física i Inorgànica, Universitat Rovira i Virgili, C/Marcel·lí Domingo 1, 43007 Tarragona, Spain; 2 Hydrogen and Power-to-X Department, Iberian Center for Research in Energy Storage (CIIAE), FUNDECYT-PCTEx, Polytechnic School of Caceres Building, Office CIIAE-C7, Av. Universidad s/n, 10003 Cáceres, Spain

**Keywords:** DFT, dimerization, fullerene, molecular dynamics, peapods

## Abstract

Fullerene dimerization inside a peapod is analyzed at DFT level by characterizing the stationary points and deriving the energy profile of the initial and reversible process named phase 1. We find that the barriers for the radical cation mechanism are significantly lower than those found for the neutral pathway. The peapod is mainly providing one-dimensional confinement for the reaction to take place in a more efficient way. Car–Parrinello metadynamics simulations provide hints on structures for the initial steps of the irreversible phase 2 where bond formation and breaking lead to important structural reorganizations within the coalescence process.

## Introduction

**Scheme 1:**
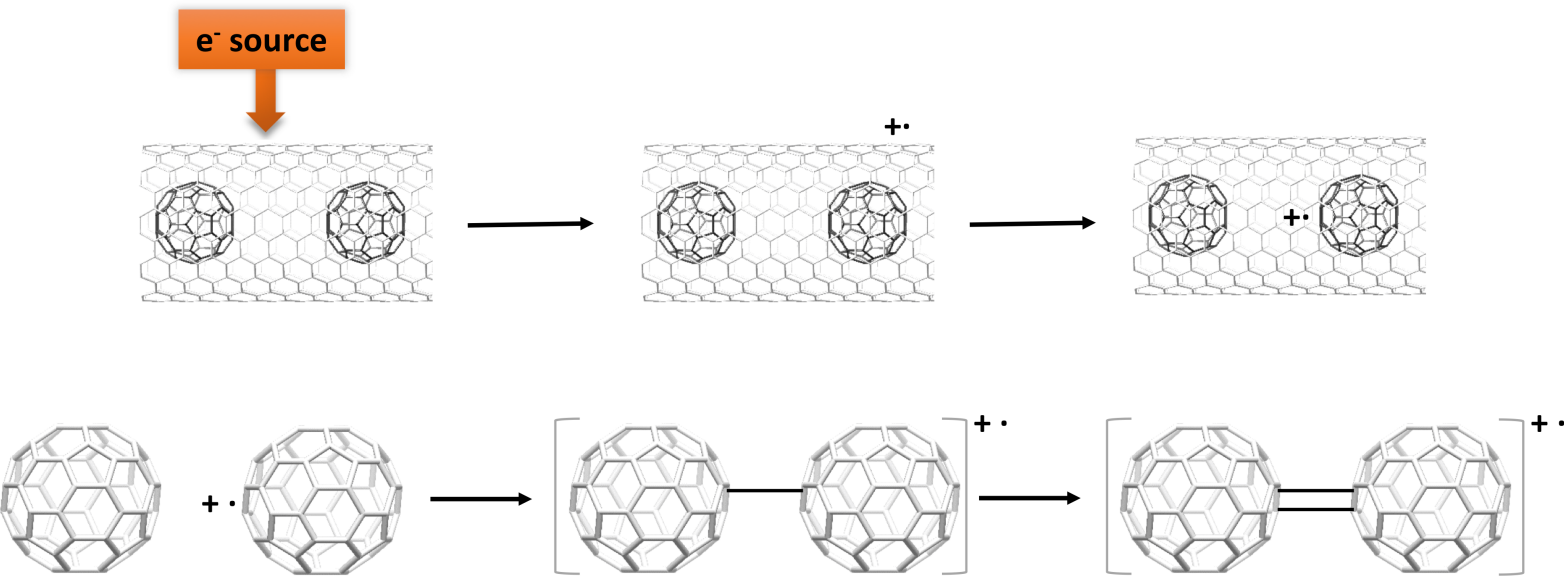
Proposed radical cation mechanism for the dimerization of two C_60_ cages inside a metallic carbon nanotube.

Transmission electron microscopy (TEM) is a technique that has been used for a long time to provide images of molecules, but also to monitor the reactions triggered by the energy transfer of the electron beam to the atoms that build the molecules. In particular, the advances in TEM as well as in methods to anchor molecules on surfaces like graphene or carbon nanotubes have allowed the scientific community to visualize at atomic resolution the structural changes of molecules in situ by single-molecule atomic-resolution real-time TEM imaging (SMART-TEM) [1–5]. Since the initial discovery [2], many movies have been published that record the dynamic behavior of a wide range of molecules and chemical reactions. One such process was the dimerization of C_60_ fullerene in a carbon nanotube peapod, i.e., hybrid structures consisting of fullerene molecules encapsulated in single-walled carbon nanotubes (SWCNT) [6–8]. Different stages of dimerization of C_60_ molecules inside a peapod, a reaction confined within a one-dimensional nanoscale space, have been detected in the last decade [3–4,7,9]. Nakamura and co-workers termed ‘phase 1’ the stage with reversible bond formation and ‘phase 2’ the stage with irreversible C–C fusions [7]. In phase 1, a [2 + 2] cycloadduct C_120_ dimer is formed, which was initially proposed to be with *C_s_* symmetry, in contrast to the X-ray structure for the C_120_ dimer that shows *D*_2_*_h_* symmetry [3,9]. In phase 2, irreversible structural rearrangements occur leading to a nanotubular-shaped fullerene inside the peapod. Kinetic analysis with the variable-temperature (VT) SMART-TEM method for the aforementioned C_60_ dimerization has also been reported by these authors [9]. They concluded that the SWCNT, which accumulates energy by the interaction with the electron beam, activates the reaction either via singlet excitation or via radical cation formation (Scheme 1). Estimation of the activation barrier for the [2 + 2] cycloaddition when the nanotube acts as a sensitizer is 33.5 ± 6.8 kJ mol^−1^. This value agrees with computational predictions for the reaction via an excited singlet state [10].

Although only a few analyses of the reaction mechanisms have been studied due to the complexity of the system, several intermediates inside the CNT have been proposed [3,11–12], which may be different from those proposed to take place in the gas phase or in the solid state at high pressures and high temperatures [13–15]. We aim to shed light in these reaction mechanisms and energy profiles by using complementary methodologies as standard density functional theory (DFT) calculations and first-principles Car–Parrinello molecular dynamics (CPMD) simulations. Firstly, we have analyzed the interaction between C_60_ and the nanotube within the peapod. Next, we have found that some dimeric C_60_–C_60_ fullerene structures inside the carbon nanotube are thermodynamically favorable. Experiments indicate that, besides C_60_ sensitization via a singlet excited state, the [2 + 2] cycloaddition can also be activated through the formation of C_60_^+•^ radical cation [3,9]. This mechanistic proposal for phase 1, which to our knowledge has not yet been explored in detail inside a carbon nanotube, is analyzed here and compared to the non-activated C_60_ dimerization. Finally, some intermediates for the subsequent irreversible C–C fusions occurring in phase 2 are proposed with the help of accelerated Car–Parrinello MD simulations.

## Results and Discussion

### Nanotube-C_60_ interaction: stabilization of the peapod

**Figure 1:**
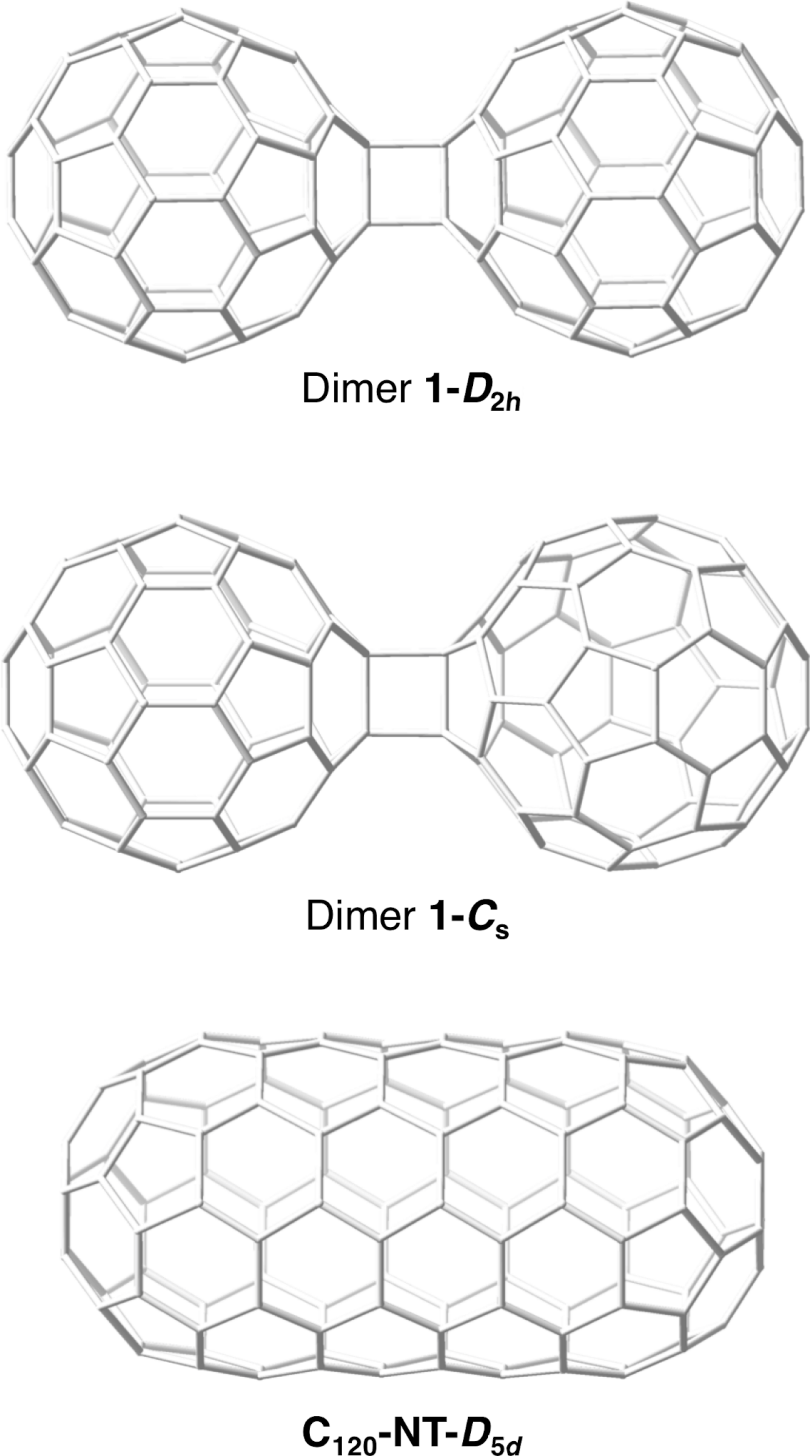
DFT-optimized structures of C_60_ dimers **1-*D*_2_*_h_***, **1-*C_s_*** and nanotubular **C_120_-NT-*D*_5_*_d_*** fullerene.

First, we estimated the size of the stabilizing interaction that holds the peapod, that is, the interaction between the C_60_ surface and the walls of the armchair (10,10) CNT. The interaction or encapsulation energy of a fullerene inside the CNT, defined as *E*_encap_ = *E*_Fuller@CNT_ – *E*_Fuller_ – *E*_CNT_, amounts to −3.23 eV for C_60_ at our present computational settings (PBE/plane waves, see section Computational methods). This significant amount of energy, which could be overestimated, comes from the π–π interactions between the C_60_ surface and the CNT wall and is modelled in a first approximation using Grimme’s corrections to the dispersion energy [16]. This interaction energy is comparable to those in similar systems with π–π interactions, as for example a “buckyball catcher” complex with C_60_ or the interlayer interaction between graphene sheets [16]. It is found to be significantly smaller than for C_60_@C_240_ [17], but larger than for C_60_@C_540_ and C_60_@C_960_ nanoonions [18]. The encapsulation energy of two separated C_60_ molecules essentially doubles that of a single molecule (−6.48 eV, see Figure S1 in Supporting Information File 1). For different C_60_ dimers (Figure 1), the interaction energies range between −6.45 and −6.50 eV (Figure S1 in Supporting Information File 1), a range which is around 1% of the total encapsulation energy. As a consequence of the effective π−π interactions, the larger the contact between the fullerene dimer surface and the CNT wall the larger the encapsulation energy. This significant interaction is also apparent from an inspection of the electronic structure of the peapod, where we can observe some molecular orbitals with non-spurious contributions from each of the CNT and C_60_ fragments (Figures S2–S4 in Supporting Information File 1).

### Relative stabilities of C_60_–C_60_ dimers

We computed the reaction energies for the dimerization of two C_60_ molecules in the one-dimensional space within the CNT and compared the results with those for the same reaction in the gas phase. We assumed that the cation radical mechanism takes place, that is, the ionized CNT generates a radical cation C_60_^•+^ that reacts with a C_60_ molecule to yield different C_120_^•+^ dimers. The energies for the dimerization of two neutral C_60_ molecules were also computed for comparison (Table 1). As products, we have considered dimer **1-*D*_2_*_h_*** and dimer **1-*C_s_***, which are products of reversible [2 + 2] cycloadditions (phase 1) between two [6,6]-bonds in the former case and, between a [6,6]-bond and a [6,5]-bond in the latter (Figure 1). Dimer **1-*C_s_*** is at our computational settings (PBE/PW), more than 15 kcal mol^−1^ higher in energy than dimer **1-*D*_2_*_h_***, the one characterized by X-ray crystallography in the solid state, both in the gas phase and inside the CNT. Similar lower stabilities for dimer **1-*C_s_*** are also found for the radical cation products, especially in the gas phase (second column in Table 1). The reaction energies are, however, notably more negative for the radical cation products. Besides, radical cations **1-*C_s_***^•^**^+^** and **1-*D*_2_*_h_***^•^**^+^** show comparable energies. Finally, for the nanotubular-shaped **C_120_-NT-*D*_5_*_d_*** isomer, the formation energies with respect to C_60_ + C_60_ are up to −300 kcal mol^−1^, which reflects the high degree of C–C bond reorganization needed to obtain this fullerene isomer that satisfies the so-called isolated pentagon rule (IPR) [19]. We corroborated our results for the gas-phase products using somewhat different computational settings with a non-periodic electronic structure code as ADF, see values in parenthesis in the two first columns of Table 1 (also at PBE level, but using atomic orbitals instead of plane waves as basis sets, see Computational methods). Although reaction energies are predicted to be somewhat more exothermic in most of the cases, relative energies between isomers are very similar, both for the neutral as well as for the radical cation dimers, what confirms the reliability of the computational settings used in the periodic VASP code.

**Table 1:** Reaction energies for the dimerization of two neutral C_60_ molecules and one neutral C_60_ and one radical cation C_60_^•+^ to yield different C_120_ dimers.^a^

	Gas-phase		@SWCNT	

	C_60_ + C_60_	C_60_ + C_60_^•+^	C_60_ + C_60_	C_60_ + C_60_^•+^

dimer **1-*D*_2h_**	−6.1	-23.6	-5.7	−8.9
	(−9.9)	(−23.4)		
dimer **1-*C*_s_**	+11.3	−10.0	+10.6	−6.6
	(+7.6)	(−10.7)		
**C** **_120_** **-NT-** ***D*** **_5d_**	−296.3	−329.3	−308.3	−332.2
	(−298.6)	(−327.5)		

^a^Energies referred to 2 C_60_ and C_60_ + C_60_^•+^ (in kcal mol^−1^) for the dimers represented in Figure 1 in the gas phase and inside the carbon nanotube (@SWCNT). Energies calculated using VASP package (PBE/PW-PAW). Energies in parenthesis were obtained with the ADF code (PBE/TZP).

### Energy profile for the reversible [2 + 2] cycloaddition in phase 1

**Figure 2:**
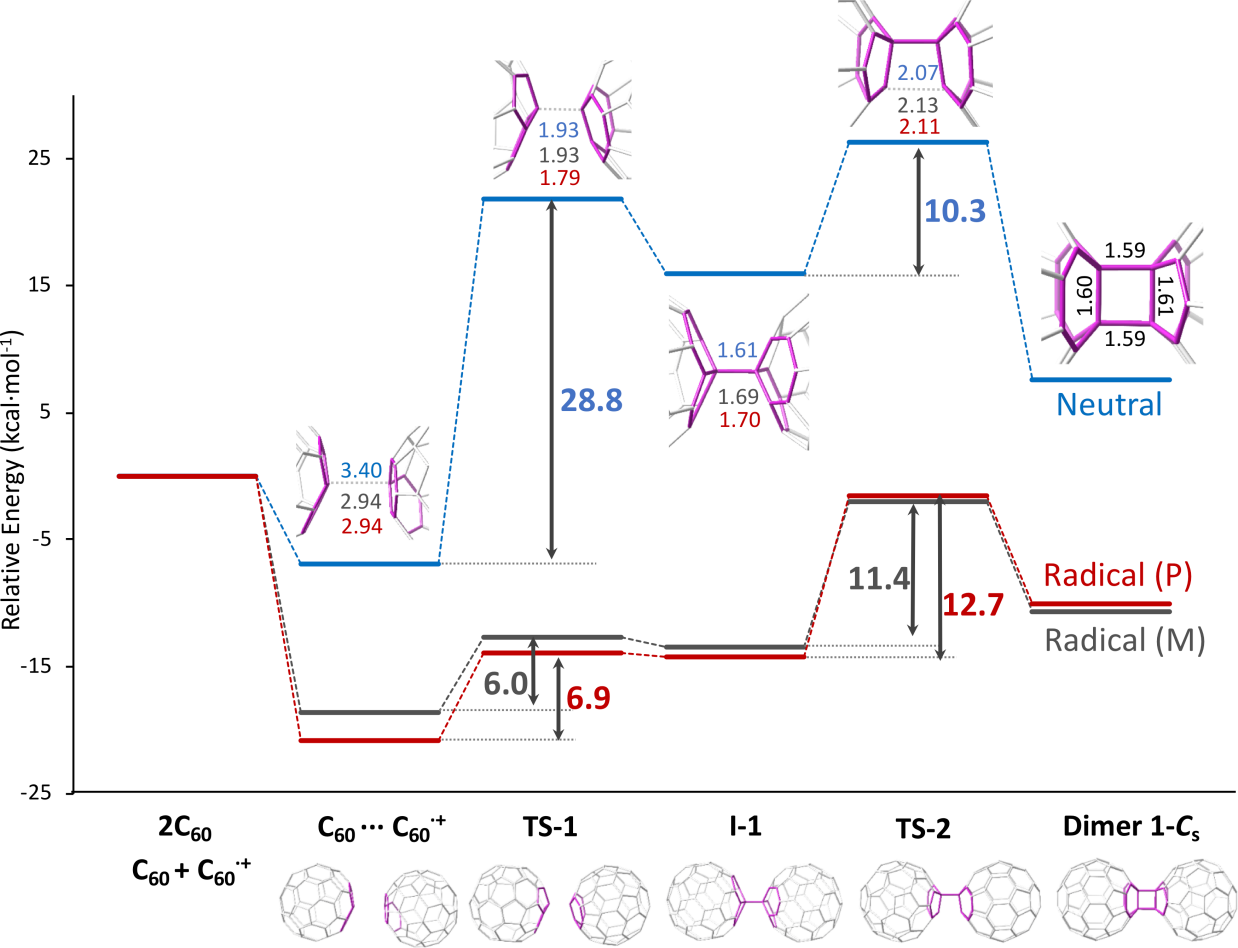
Energy profiles for the dimerization of 2 C_60_ and C_60_ + C_60_^•+^ fullerenes in the gas phase. All energy barriers are in kcal mol^−1^ and distances of the most relevant bonds for the different structures are represented in Å in a zoomed image. Energy profile for C_60_ + C_60_^+•^ radical dimerization (phase 1) computed with the periodic (P) approximation (plane waves, VASP) is represented in red, while energy profiles for C_60_ + C_60_^+•^ radical and C_60_ + C_60_ neutral dimerizations computed with the standard molecular (M) approach (atomic basis functions, ADF) are represented in grey and blue, respectively.

We analyzed in detail the energy profile for the first step in the dimerization process, that is, the reversible [2 + 2] cycloaddition to obtain dimers **1-*C_s_*** and **1-D_2_*_h_***. We initially considered dimerization in the gas phase to check the reliability of our methodology (periodic calculations using VASP code) by comparing with other more standard procedures (non-periodic calculations using ADF code; see Computational methods for details) when computing the energy profile for the radical cation C_60_ + C_60_^+^. Besides, we computed the profile for the neutral dimerization C_60_ + C_60_ to assess the effect ionization produced by the electron beam has in the process. Before the reaction takes place, a stabilizing van der Waals complex between the two C_60_ molecules was formed (Figure 2), with an interdimer distance around 3 Å (slightly larger for the neutral profile). For dimer **1-*C_s_***, the stabilization of this complex is significantly more important for the radical cation (around 20 kcal mol^−1^) than for the neutral complex (less than 10 kcal mol^−1^). To reach intermediate **I-1**, the singly-bonded dimer, a transition state **TS-1** has to be overcome. The barrier for the radical cation is much smaller (6–7 kcal mol^−1^) than for the neutral dimer (28.8 kcal mol^−1^), so we confirm that the process is activated for the cation. The interdimer C···C distance is smaller than 2 Å for the radical cation and for the neutral species. Once intermediate **I-1** is formed, the interdimer C–C distance is around 1.60–1.70 Å. Formation of the second C–C bond to yield dimer **1-*C_s_*** requires to overcome a second transition state **TS-2** with energy barriers that range between 10–13 kcal mol^−1^ from the immediate intermediate depending on the profile. The interdimer C···C distance of the forming bond is slightly larger than 2 Å in all three cases. Finally, formation of dimer **1-*C_s_*** is exothermic for the radical cation profile, but moderately endothermic for the neutral dimer, as previously observed. As general trends, we find that the radical cation profiles are qualitatively parallel with some minimal differences, which corroborates the validity of our periodic approximation to study the dimerization of the radical cation inside the CNT (see below). The rate-determining transition state for the radical cation and for the neutral dimer is the same (TS2) as well as the lowest-energy intermediate (van der Waals complex), so the two profiles are not that different, according to the energetic spam model by Kozuch and Shaik [20]. In any case, the mechanism via radical cation is the most favorable, both kinetically and thermodynamically. For dimer **1-D_2_*_h_***^•^**^+^**, the energy profile up to **I-1** is very similar to that of dimer **1-C*_s_***^•^**^+^** (see Supporting Information File 1, Figure S9). However, **TS-2** is significantly lower in energy. Therefore, in gas phase, dimer **1-*D*_2_*_h_***^•^**^+^** is predicted to be the thermodynamic and the kinetic product.

**Figure 3:**
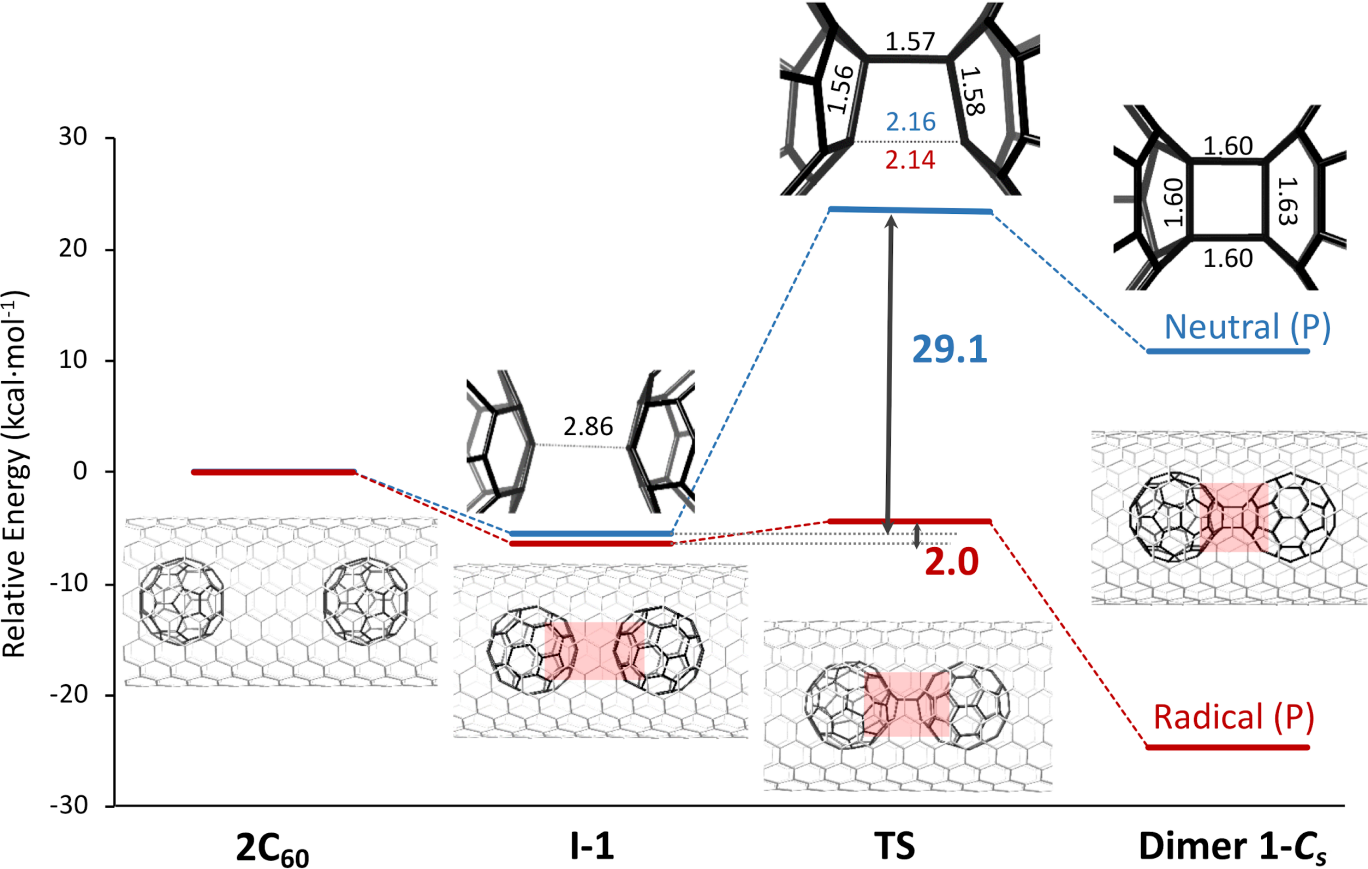
Energy profiles for the dimerization of 2 C_60_ (neutral) and C_60_ + C_60_^•+^ (radical cation) fullerenes inside the SWCNT computed with the periodic (P) approximation (plane waves, VASP). Energy barriers are in kcal mol^−1^ and distances of the most relevant bonds for the different structures are in Å.

Once our methodology was validated, the energy profile for the formation of dimer **1-*C*_s_** inside the CNT was analyzed. In contrast to the gas phase reaction, we now only found the initial van der Waals complex and a single transition state **TS** for both the neutral and the radical cation profiles (Figure 3). The van der Waals complex shows an interdimer C···C distance of 2.86 Å, slightly shorter than the one observed for the gas phase reaction. The transition state **TS** corresponds now to the formation of the second interdimer C···C distance, around 2.15 Å (Figure 3), once the first C–C bond is already formed. All the attempts to obtain an intermediate with a single C–C interdimer bond failed. The energy barrier to overcome the **TS** is predicted to be 29.1 kcal mol^−1^ for the neutral dimer, but only 2.0 kcal mol^−1^ for the radical cation. Therefore, the first step of the C_60_ dimerization is significantly faster for the radical cation than for the neutral system. Formation of dimer **1-*C_s_***@CNT is appreciably endothermic for the neutral profile (>10 kcal mol^−1^), but fairly exothermic (−25 kcal mol^−1^, Figure 3) for the radical cation dimer (C_60_-C_60_)^•+^@CNT. Albeit some differences are present, the general features of the energy profiles inside the CNT are not that different from those in the gas phase; the C_60_ + C_60_ dimerization is slightly endothermic with a barrier around 30 kcal mol^−1^, whereas dimerization for the radical cation is exothermic with a tiny (or almost nil) barrier, which makes this initial step of the reaction drastically faster. Therefore, the main function of the CNT is to *constrain* the translation of C_60_ molecules to a one-dimensional space to maximize the rate of collisions between them. Finally, we would like to point out that in the reversible phase 1, radical cation (**1-*D*_2_*_h_***)^•+^@CNT, which shows slightly lower energy than (**1-*C_s_***)^•+^@CNT (2 kcal mol^−1^ with our settings) is also predicted to be formed. Free energy difference between the two isomers, estimated in the gas phase, is somewhat reduced when increasing temperature (see Supporting Information File 1, Table S1). Therefore, the molar fraction of (**1-*C_s_***)^•+^@CNT would increase slightly at higher temperatures.

### Mechanistic insights on the irreversible phase 2: first steps

**Figure 4:**
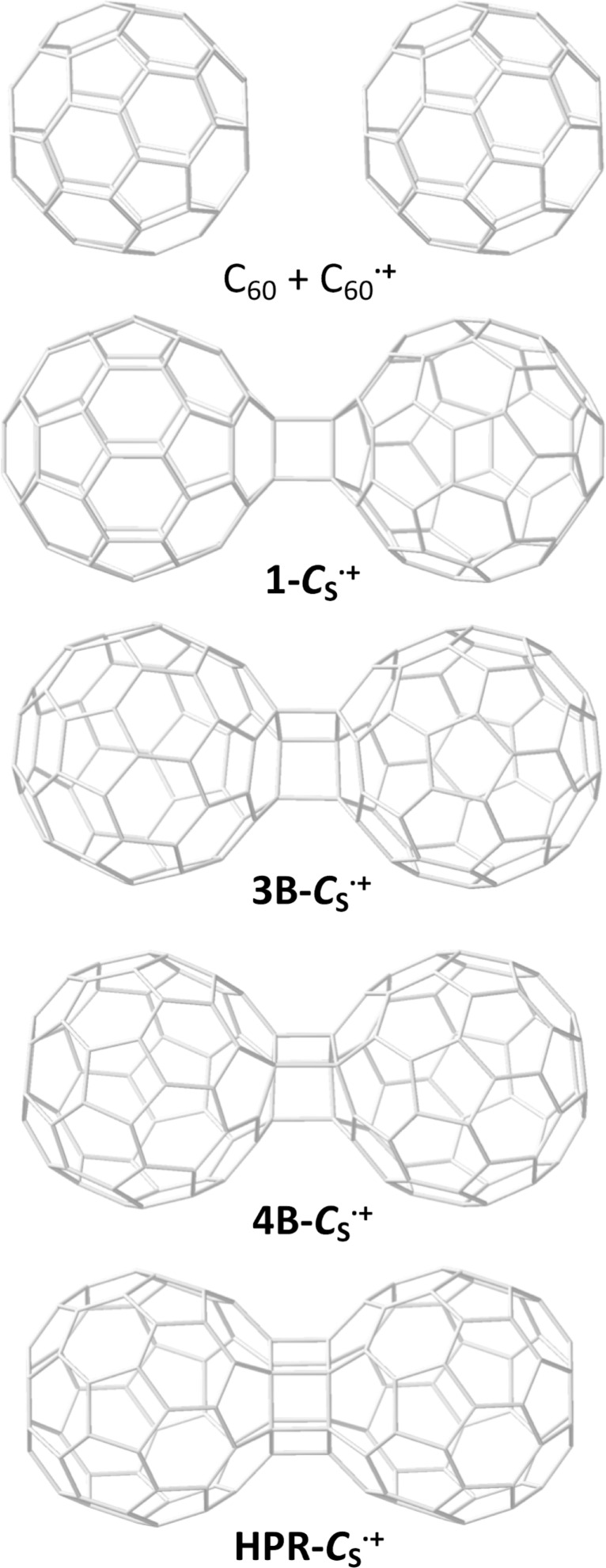
Proposed sequence of C_60_ dimers up to the formation of dimer **HPR-*C_s_***^•^**^+^**.

Once phase 1 was analyzed in detail, we studied the irreversible formation of C–C bonds in the interdimer region, known as phase 2 in the dimerization process. Due to the large number of possible pathways to be investigated, we made use of a simulation technique that allowed us to explore the free energy surface in a fast and efficient way. In particular, we performed Car–Parrinello metadynamics simulations by choosing collective variables (CVs) that describe the formation and breaking of C–C bonds in the interdimer region (see Computational methods for more details). It is not the goal of the present work to explain all the steps up to the formation of fullertube C_120_, but the steps that follow the formation of dimers **1-*C_s_***^•^**^+^** and **1-*D*_2_*_h_***^•^**^+^**. We first run a metadynamics simulation for dimer **1-*C_s_***^•^**^+^** using as CV the coordination number of nine carbon atoms of one C_60_ molecule (those of contiguous hexagon and pentagon) with respect to nine carbon atoms in the other C_60_, all of them in the interdimer region (see Supporting Information File 1). Choosing this single CV, we aim to rapidly and efficiently explore the region of the free energy surface that describes the formation of irreversible C–C bonds. Molecular dynamics simulations were done in the radical C_120_ dimer alone; we did not consider the interaction with the CNT. After a 4 ps metadynamics, we observed the sequential formation of C–C bonds between the two C_60_ cages up to a number of six in structures that we have called dimer **3B-*C_s_***^•^**^+^** (three bonds), dimer **4B-*C_s_***^•^**^+^** (four bonds), dimer **5B-*C_s_***^•^**^+^** (five bonds) and finally dimer **HPR-*C_s_***^•^**^+^** (six bonds, see Figure 4). In the latter, one hexagon from each C_60_ face each other with six interdimer bonds formed between the C atoms at the vertexes of each hexagon forming a hexagonal prism (HPR). Several authors have already proposed this HPR structure for the dimerization of two neutral C_60_ cages [13–15]. We characterized this structure as a minimum of the potential energy surface at 49.2 kcal mol^−1^ (PBE/TZP) higher than dimer **1-*C_s_***^•^**^+^** (Table 2). The C–C distances of the six new bonds are 1.597 Å and the six C–C distances within each hexagon have been elongated from 1.41–1.49 in C_60_ to 1.55–1.57 Å (Table S2). Dimers **3B-*C_s_***^•^**^+^** and **4B-*C_s_***^•^**^+^** are also characterized as minima at 18.7 and 34.3 kcal mol^−1^ with respect to dimer **1-*C_s_***^•^**^+^** (Table 2). Structure of dimer **5B-*C_s_***^•^**^+^** found in the metadynamics, however, is not a minimum and leads to dimer **3B-*C_s_***^•^**^+^** upon geometry optimization. C–C distances of the new bonds are within the range 1.53–1.71 Å. For dimer **1-*C_s_***^•^**^+^**, the two distances are equivalent (1.587 Å) as they are the six distances for **HPR-*C_s_***^•^**^+^** (1.597 Å); for dimers **3B-*C_s_***^•^**^+^** and **4B-*C_s_***^•^**^+^**, however, more asymmetry in the distances is found. Similar C–C distances within the interacting hexagons exist, all of them within the range 1.55–1.60 Å (Supporting Information File 1, Table S2). A second metadynamics run for dimer **1-*D*_2_*_h_***^•^**^+^** was also done using as CV the coordination number of ten carbon atoms of one C_60_ molecule (two contiguous hexagons) with respect to ten carbon atoms in the other C_60_, analogous to the first run. After 7 ps, we also observed the sequential formation of C–C bonds between the two cages in structures now called dimer **3B-*D*_2_*_h_***^•^**^+^** (three bonds), dimer **4B-*D*_2_*_h_***^•^**^+^** (four bonds), dimer **5B-*D*_2_*_h_***^•^**^+^** (five bonds) and finally dimer **HPR-*D*_2_*_h_***^•^**^+^** (six bonds, see Figure S10). Dimers **3B-*D*_2_*_h_***^•^**^+^**, **4B-*D*_2_*_h_***^•^**^+^** and **HPR-*D*_2_*_h_***^•^**^+^** are characterized as minima at 32.7, 39.3 and 63.3 kcal mol^−1^ with respect to dimer **1-*D*_2_*_h_***^•^**^+^** (Table 2). The C–C bond distances are very similar to those of the corresponding ***C_s_*** dimers (Supporting Information File 1, Table S2).

**Table 2:** Relative energies for several dimers during the reaction of one neutral C_60_ and one radical cation C_60_^•+^.^a^

	***C*** ***_s_*** ^•+^	***D*** **_2_** ***_h_*** ^•^ **^+^**

dimer **1**	12.7	0.0
dimer **3B**^•^**^+^**	31.4	32.7
dimer **4B**^•^**^+^**	47.0	39.3
dimer **HPR**^•^**^+^**	61.9	63.3

^a^Relative energies with respect to dimer **1-*D*_2_*_h_***^•^**^+^** (in kcal mol^−1^). Corresponding dimers are shown in Figure 4 and Supporting Information File 1, Figure S10.

## Conclusion

Two different computational methodologies, molecular approach versus periodic solid-state approach provide analogous and consistent results, which gives strong support and reliability to the predictions. In general, the reaction of two C_60_ molecules to yield different dimers is exothermic and the exothermicity is enhanced when radical cation dimers are formed. The thermodynamics of dimerization inside the nanotube is not that different as found in the gas phase; the nanotube mainly provides a 1D confinement for the reaction to proceed. The barriers for the reversible phase 1 are found to be easily surmountable at ambient temperature for both the forward and the inverse processes, especially for the dimerization of radical cation fullerenes. For the initial steps of irreversible phase 2, up to six C–C bonds in the interdimer region are formed leading to a hexagonal prism shared by the two cages, with significantly larger barriers than in phase 1, and the processes, consequently, slower. Next steps of radical cation dimerization inside the CNT (phase 2), which are under study, combined with new SMART-TEM images and movies will provide us a deeper understanding of fullerene coalescence processes in peapods.

## Computational Methods

The Amsterdam Density Functional (ADF) code [21–22] was used for the electronic structure calculations and to optimize reactants, products, intermediates and transition states. The Perdew, Burke and Ernzerhoff (PBE) functional provided the electronic density [23]. Electrons were described with Slater-type basis functions of triple-ζ + polarization quality. We have included scalar relativistic corrections using the zeroth-order regular approximation (ZORA) formalism. Grimme3 BJ-DAMP dispersion corrections have also been included in all calculations [24]. Stationary points were fully characterized by computing the Hessian matrix.

Car–Parrinello molecular dynamics (CPMD) simulations were performed by means of the CPMD program [25–26]. The description of the electronic structure was based on the expansion of the valence electronic wave functions in a plane wave (PW) basis set, which was limited by an energy cutoff of 40 Ry. The interaction between the valence electrons and the ionic cores was treated through the pseudopotential (PP) approximation (Martins–Troullier type) [27]. The PBE functional was selected as the density functional. Dispersion corrections were also considered in the calculations. We used a fictitious electron mass of 800 a.u. The simulations were carried out using periodic boundary conditions in a tetragonal cell (side length of 11 Å and height of 20 Å) and a time step of 0.144 fs. We used the metadynamics technique to analyze the dimerization reaction mechanism [28–30]. The collective variable (CV) considered for the exploration of the free-energy surface was the coordination number of nine C atoms of one C_60_ (those that are involved in a hexagon fused with a pentagon) with respect to the nine C atoms (hexagon-pentagon) on the other C_60_ molecule (see Supporting Information File 1 for details). The armchair-type single-wall carbon nanotube (SWCNT) (10,10) with a length of 22.14 Å and a diameter of 13.65 Å was employed in the calculations.

The fullerene dimerization mechanism inside a SWCNT was also studied by density functional theory applied to periodic systems through VASP code [31]. The exchange-correlation functional used was PBE with the zero damping DFT-D3 method of Grimme et al. [16]. Inner electrons were replaced by PAW pseudopotentials [16] while valence electron density was expanded in plane waves with a maximum kinetic energy of 400 eV. The model of SWCNT was 29.61 Å long and with a diameter of 13.65 Å, embedded in a box of 30 × 30 Å of vacuum in the plane perpendicular to the nanotube axis. The gas-phase structures were in a cubic box of 30 Å side. The k-point sampling was performed through the Gamma–Pack scheme with one point. Transition states were obtained through the climbing image version of the nudged elastic band algorithm [32] and dimer method [33]. These structures showed a single imaginary frequency or with some negligible imaginary frequencies (under 10 cm^−1^). The projected density of states was calculated and own code estimated the bands that belong to fullerene or SWCNT. The excited states of the fullerene dimer inside the SWCNT were studied with a single-point energy calculation of a given band structure where one electron of the highest-occupied band of the fullerene is excited to the lowest-unoccupied band of SWCNT.

## Supporting Information

File 1 Additional details of the computational settings and results: encapsulation energies, molecular orbitals (MO), MO diagrams, intermediate structures in metadynamics and optimized xyz coordinates for the dimers.
