# Supplementary material for: Electron-beam-promoted fullerene dimerization in nanotubes: insights from DFT computations
Source: Beilstein J Org Chem. 2024 Jan 17;20:92–100. doi: 10.3762/bjoc.20.10 (PMC10804564; doi:10.3762/bjoc.20.10)
Supplement: File 1 — Additional details of the computational settings and results: encapsulation energies, molecular orbitals (MO), MO diagrams, intermediate structures in metadynamics and optimized xyz coordinates for the dimers. [file Beilstein_J_Org_Chem-20-92-s001.pdf]

BEILSTEIN JOURNAL OF ORGANIC CHEMISTRY

Supporting Information

for

Electron-beam-promoted fullerene dimerization in nanotubes: insights from DFT computations

Laura Abella, Gerard Novell-Leruth, Josep M. Ricart, Josep M. Poblet
and Antonio Rodríguez-Fortea

Beilstein J. Org. Chem. **2024,** *20,* 92–100. doi:10.3762/bjoc.20.10

Additional details of the computational settings and results: encapsulation energies, molecular orbitals (MO), MO diagrams, intermediate structures in metadynamics and optimized xyz coordinates for the dimers

License and Terms: This is a supporting information file under the terms of the Creative Commons Attribution License (https://creativecommons.org/licenses/by/4.0). Please note that the reuse, redistribution and reproduction in particular requires that the author(s) and source are credited and that individual graphics may be subject to special legal provisions.
The license is subject to the *Beilstein Journal of Organic Chemistry* terms and conditions: (https://www.beilstein-journals.org/bjoc/terms)

FULLERENE@CARBON NANOTUBE (X@SWCNT)

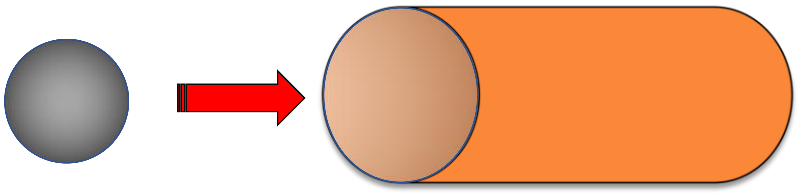

Encapsulation energy of fullerenes inside CNT

$$E_{int} = E_{Cxx@CNT} - E_{CNT} - E_{Cxx}$$

**(X@SWCNT)**	eV
C_{60}	-3.23
$C_{60}\cdots C_{60}$	-6.48
Dimer **1-D_{2h}**	-6.45
Dimer **1-C_s**	-6.50

Figure S1: Encapsulation energies (in eV) of C_{60} and C_{60} dimers inside the carbon nanotube (CNT).

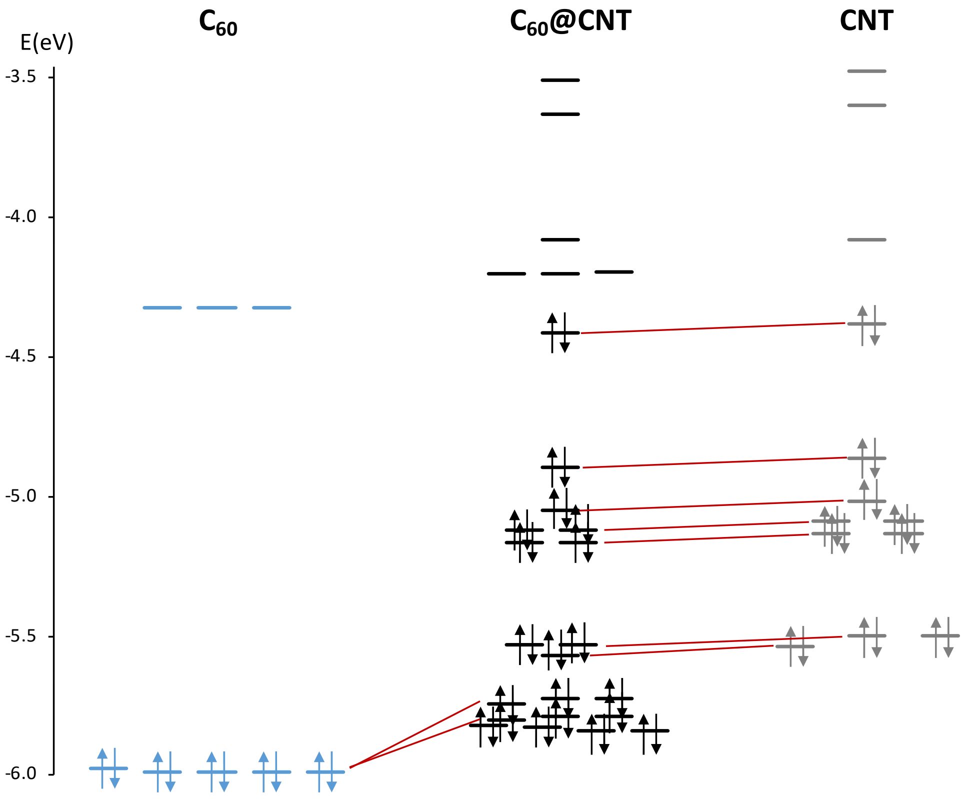

Figure S2: Molecular orbital diagram of the C_{60}@CNT peapod as a combination of molecular fragments of one C_{60} fullerene and the carbon nanotube (CNT) using the ADF/PBE/TZP/D3-BJDAMP level of theory.

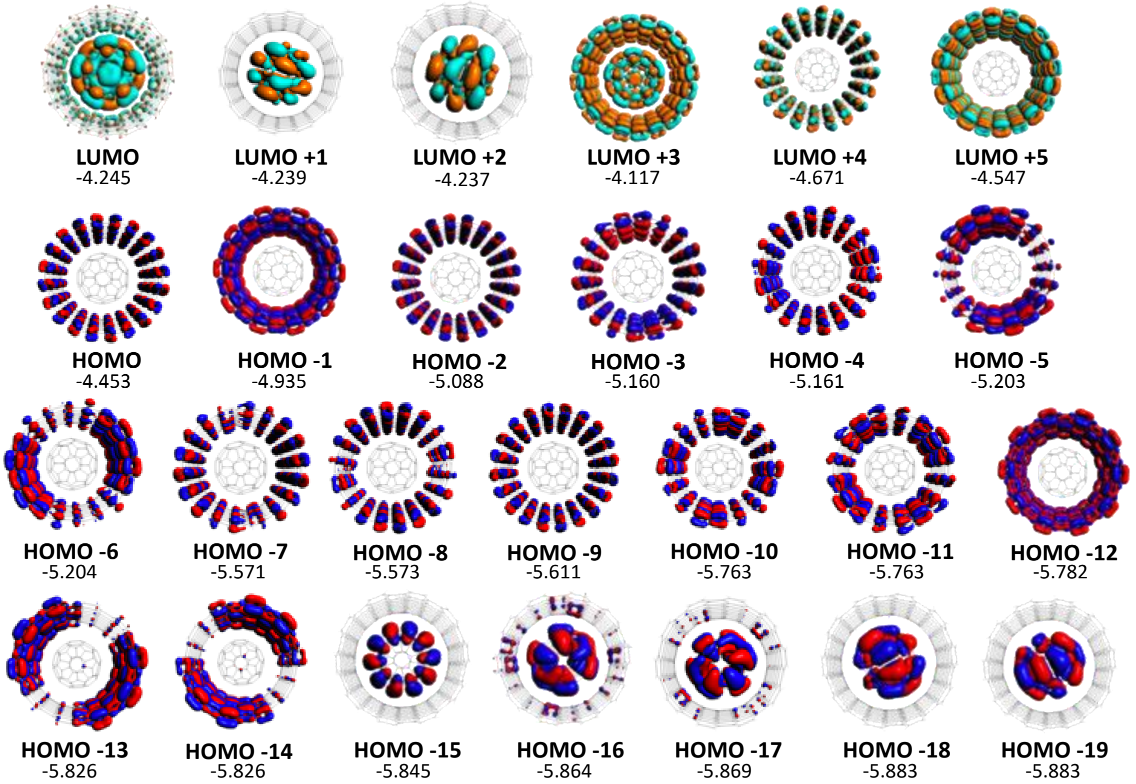

Figure S3: Isosurfaces (±0.01) of the C_{60}@CNT peapod with the corresponding molecular orbital energy in eV using the ADF/PBE/TZP/D3-BJDAMP level of theory.

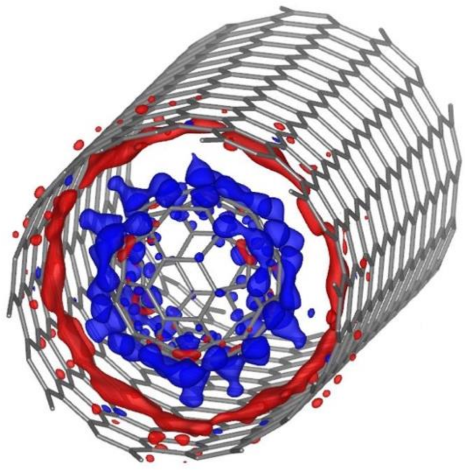

Figure S4: Difference charge density of the C_{60}@CNT peapod defined as $\Delta\rho = \rho_{C60@CNT} - \rho_{CNT} - \rho_{C60}$. There is a small electron transfer of 0.02 e from the CNT to the fullerene.

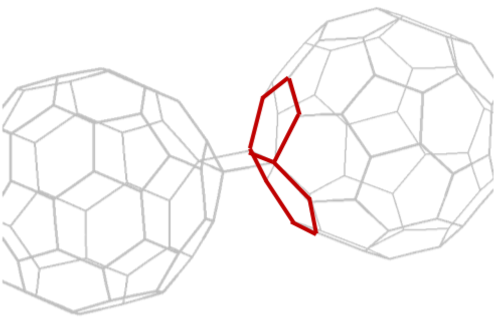

Figure S5: The highlighted carbon atoms are those used to define the coordination number used as collective variable in the metadynamics simulation starting from dimer **1-*C*s**$^{\cdot+}$. The coordination number considered is that of the nine highlighted C atoms of a C_{60} molecule with respect to the nine equivalent C atoms of the second C_{60} molecule.

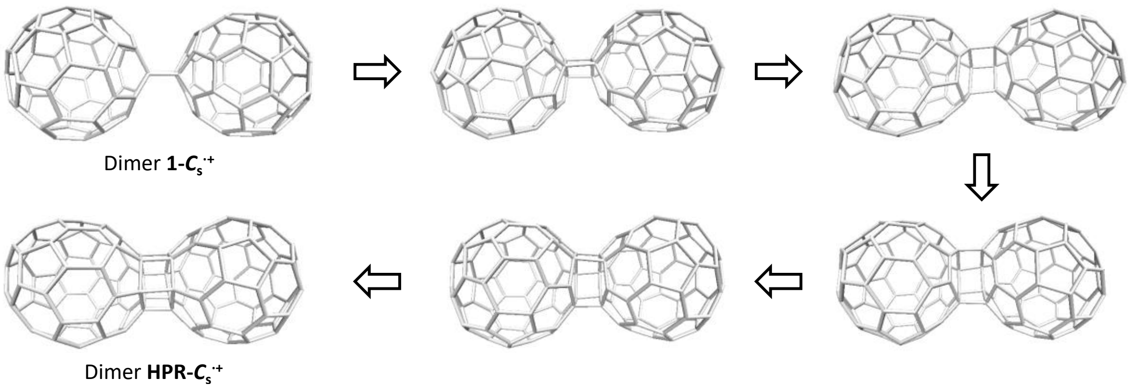

Figure S6: Structures observed in the metadynamics run at 793 K (4 ps) starting from dimer **1-*C*s**$^{\cdot+}$. The number of C–C bonds increases from two in dimer **1-*C*s**$^{\cdot+}$ to six in dimer **HPR-*C*s**$^{\cdot+}$.

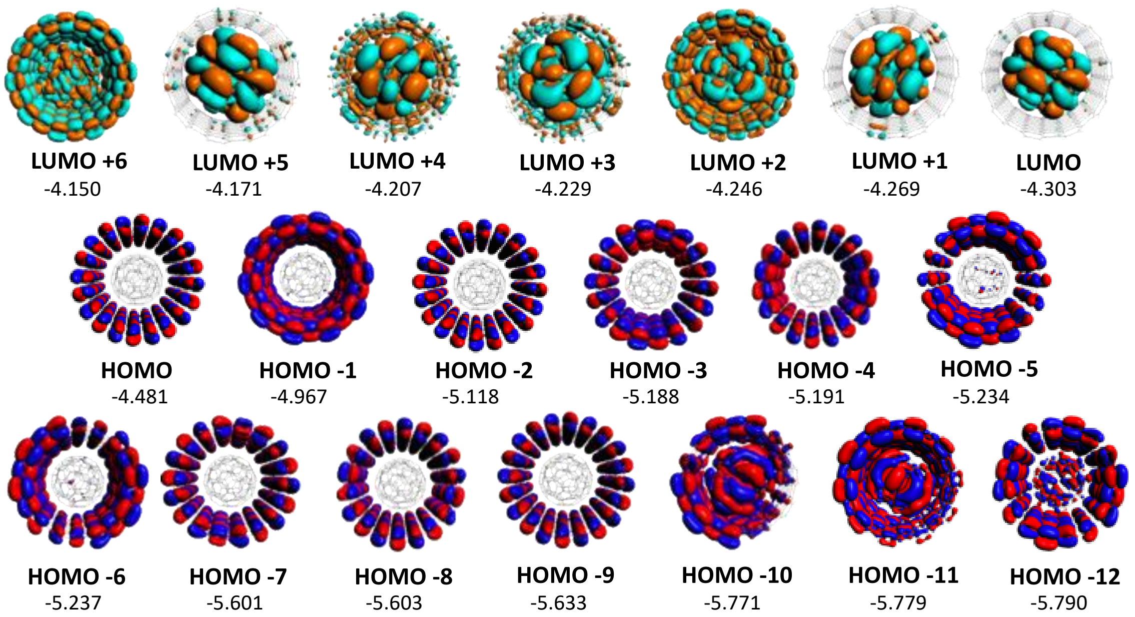

Figure S7: Isosurfaces (±0.005) of selected molecular orbitals (MOs) for the DFT-optimized structure of $(C_{60})_2$@CNT. Energies (in eV) for each orbital are indicated.

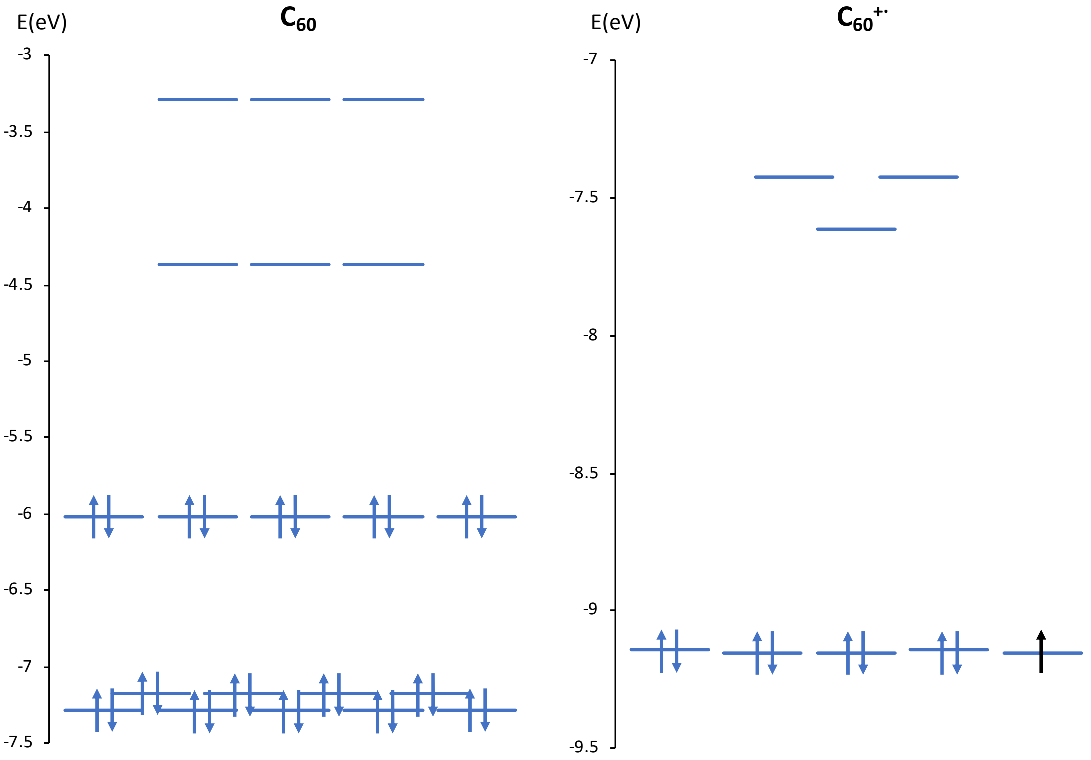

Figure S8: Representation of the molecular diagrams for C_{60} and $C_{60}^{+\cdot}$ fullerenes. Energies for each orbital in eV.

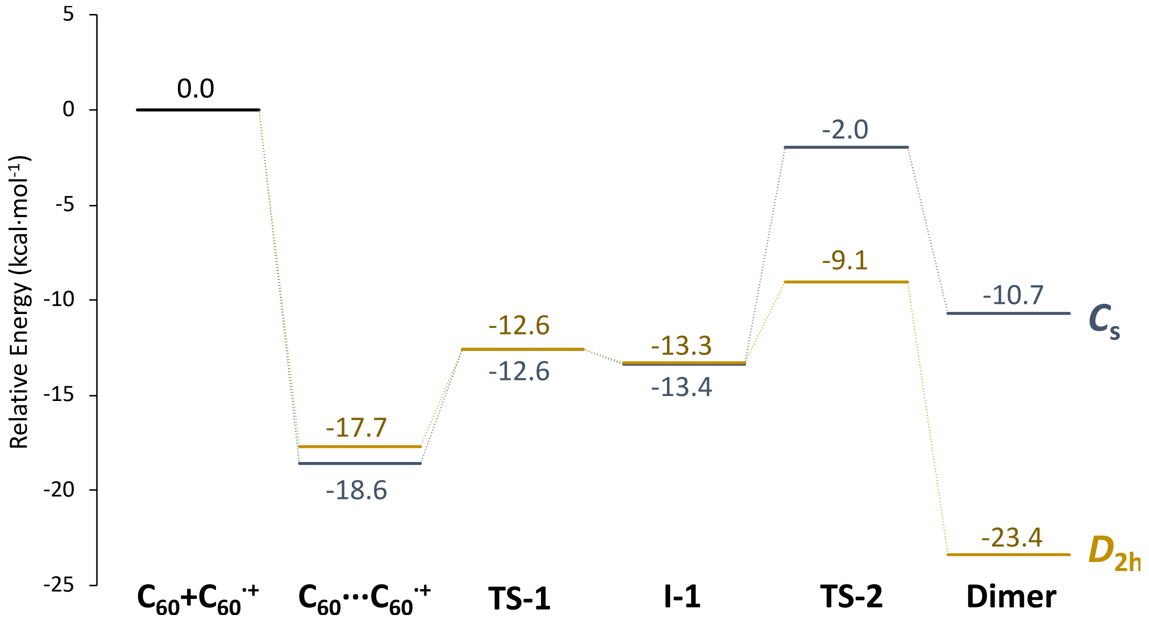

Figure S9: Energy profiles for the dimerization of C_{60} + $C_{60}^{\cdot+}$ in the gas phase, comparison of the two isomers dimer **1-$\boldsymbol{C_s}$**$^{\cdot+}$ and dimer **1-$\boldsymbol{D_{2h}}$**$^{\cdot+}$ (using ADF code, PBE/TZP). All energy values are in kcal·mol^{-1} relative to reactants. In gas phase, dimer **1-$\boldsymbol{D_{2h}}$**$^{\cdot+}$ is the thermodynamic and the kinetic product. From the energy profile inside the CNT (Figure 3), we expect that the TS for radical **1-$\boldsymbol{D_{2h}}$** would have rather similar energy as for radical **1-$\boldsymbol{C_s}$** and this last step is expected to be reversible at moderate and high temperatures, so the two isomers are expected to be formed inside the CNT according to the proposed mechanism.

Table S1: Gas phase relative energies and Gibbs free energies for radical cation dimers **1-C_s**$^{\cdot+}$ and **1-D_{2h}**$^{\cdot+}$.[a]

	Energy	Free energy 298 K	Free energy 400 K	Free energy 800 K
**1-D_{2h}**$^{\cdot+}$	0.0	0.0	0.0	0.0
**1-C_s**$^{\cdot+}$	13.2	12.3	12.0	11.0

[a] Relative energies and free energies in kcal·mol^{-1}. Free energies estimated within the harmonic and rigid rotor approximation.

Table S2. Relative energies and C–C distances for several dimers during the reaction of one neutral C_{60} and one radical cation $C_{60}^{\cdot+}$.[a]

	Rel. energy	C–C dist New bonds	C–C dist Hexagons
Dimer **1-C_s**$^{\cdot+}$	0.0	1.587	1.60-1.60
Dimer **3B-C_s**$^{\cdot+}$	18.7	1.53-1.66	1.56-1.58
Dimer **4B-C_s**$^{\cdot+}$	34.3	1.56-1.71	1.55-1.57
Dimer **HPR-C_s**$^{\cdot+}$	49.2	1.597	1.55-1.57
Dimer **1-D_{2h}**$^{\cdot+}$	0.0	1.588	1.596
Dimer **3B-D_{2h}**$^{\cdot+}$	32.7	1.53-1.65	1.57-1.58
Dimer **4B-D_{2h}**$^{\cdot+}$	39.3	1.57-1.69	1.56-1.56
Dimer **HPR-D_{2h}**$^{\cdot+}$	63.3	1.60-1.60	1.56-1.57

[a] Relative energies with respect to dimer **1-C_s**$^{\cdot+}$ and dimer **1-D_{2h}**$^{\cdot+}$ (in kcal·mol^{-1}); C–C distances (in Å) for the formed bonds in the dimer (new bonds) and within those hexagonal faces of each C_{60} that are interacting (hexagons).

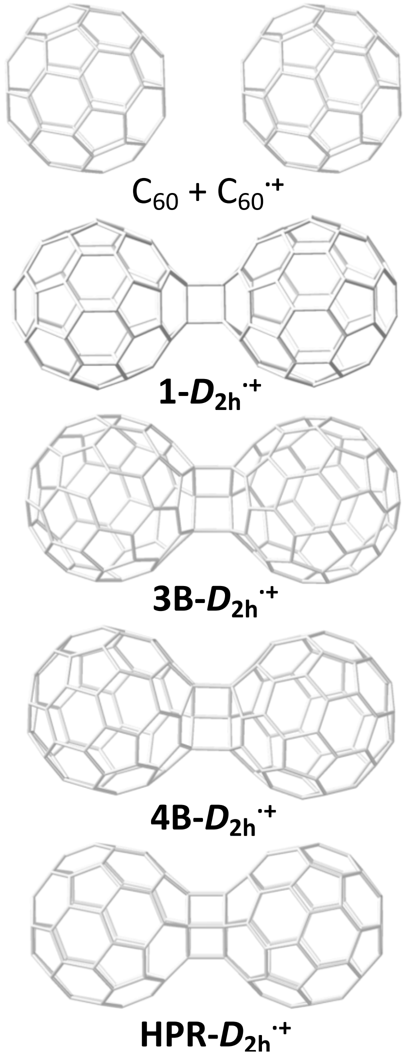

Figure S10: Proposed sequence of C_{60} dimers up to the formation of dimer **HPR-$D_{2h}^{\cdot+}$**.

xyz coordinates

Dimer **1**-$D_{2h}^{\cdot+}$

C	-3.710162	3.867641	-11.265162
C	-5.139645	3.877435	-11.002691
C	-5.463631	2.689086	-10.239747
C	-4.230741	1.940264	-10.028518
C	-3.150783	2.669495	-10.662266
C	-2.988035	5.062238	-11.245300
C	-3.665110	6.316830	-10.962304
C	-5.041098	6.327666	-10.705013
C	-5.792561	5.084407	-10.725570
C	-6.793125	5.145799	-9.676803
C	-7.109611	3.998792	-8.944398
C	-6.431286	2.745509	-9.232509
C	-6.198073	2.067059	-7.971783
C	-5.011013	1.345468	-7.768446
C	-4.012103	1.275797	-8.818326
C	-2.701770	1.321426	-8.189539
C	-1.662345	2.029501	-8.798113
C	-1.889381	2.713724	-10.057187
C	-1.137551	3.956978	-10.036547
C	-1.675970	5.108847	-10.621846
C	-1.542097	6.387961	-9.954608
C	-2.775116	7.136859	-10.165762
C	-3.290114	7.942386	-9.145841
C	-4.719187	7.954474	-8.878506
C	-5.576316	7.159398	-9.643460
C	-6.655686	6.426344	-9.001635
C	-6.825935	6.506303	-7.617974
C	-7.150280	5.315441	-6.853737
C	-7.296192	4.086272	-7.507417
C	-6.736937	2.894363	-6.911234
C	-6.060680	2.988415	-5.686371
C	-4.829916	2.240900	-5.475680
C	-4.320192	1.426289	-6.497317
C	-2.897702	1.415267	-6.754156
C	-2.052447	2.219595	-5.980647
C	-0.970180	2.949877	-6.615044
C	-0.774058	2.854079	-7.994485
C	-0.444653	4.042874	-8.764643
C	-0.309635	5.277198	-8.123628
C	-0.870300	6.473078	-8.731818
C	-1.412431	7.298930	-7.669507
C	-2.599493	8.020586	-7.872677
C	-3.597920	8.087884	-6.824497
C	-4.904384	8.044103	-7.441363
C	-5.934197	7.328021	-6.819984
C	-5.698899	6.651901	-5.559276
C	-6.461799	5.390337	-5.580044
C	-5.953841	4.240689	-4.995396
C	-4.709915	4.237838	-4.119669
C	-3.957265	3.029145	-4.655074
C	-2.596178	3.043704	-4.918762
C	-1.833464	4.304937	-4.897628
C	-0.835634	4.231786	-5.946978
C	-0.504899	5.372357	-6.688160
C	-1.180712	6.619734	-6.410688
C	-2.153845	6.667962	-5.401727
C	-3.384759	7.415498	-5.612398
C	-4.453706	6.721554	-4.954680
C	-3.886567	5.600127	-4.097500
C	-2.457898	5.509341	-4.613273
C	-4.915864	4.336241	-2.552864
C	-6.342842	4.425453	-2.032684
C	-6.643886	3.266488	-1.243889
C	-5.411802	2.519582	-1.037354
C	-4.345128	3.214909	-1.697402
C	-4.093129	5.699014	-2.531300
C	-4.845226	6.907948	-1.994992
C	-6.204778	6.891875	-1.726901
C	-6.966327	5.629556	-1.745799
C	-7.961313	5.701172	-0.693848
C	-8.289412	4.559936	0.047564
C	-7.613898	3.313193	-0.232069
C	-7.378551	2.633123	1.025354
C	-6.190380	1.912267	1.225073
C	-5.194867	1.846424	0.174136
C	-3.886728	1.891244	0.787574
C	-2.858962	2.608419	0.163898
C	-3.098126	3.285048	-1.096048
C	-2.336415	4.547243	-1.076231
C	-2.847764	5.696979	-1.658210
C	-2.739069	6.949028	-0.968311
C	-3.970739	7.696184	-1.176097
C	-4.478024	8.509221	-0.152141
C	-5.899661	8.519201	0.108478
C	-6.746069	7.714437	-0.662903
C	-7.826136	6.982767	-0.025968
C	-8.018515	7.077453	1.353954
C	-8.344678	5.888405	2.124643
C	-8.480413	4.654302	1.483540
C	-7.917566	3.458386	2.089579
C	-7.242475	3.543525	3.310565
C	-6.008387	2.795449	3.517923
C	-5.496255	1.990081	2.496641
C	-4.067766	1.979479	2.225348
C	-3.209092	2.775443	2.987888
C	-2.131461	3.509534	2.343733
C	-1.965073	3.430365	0.959530
C	-1.643670	4.621721	0.195071
C	-1.496402	5.850859	0.848734
C	-2.058861	7.042644	0.254755
C	-2.595116	7.869032	1.317046
C	-3.783844	8.588894	1.117202
C	-4.779790	8.656897	2.169678
C	-6.091723	8.611453	1.544650
C	-7.129006	7.902575	2.155767
C	-6.897420	7.217818	3.413561
C	-7.648075	5.974305	3.394447
C	-7.107098	4.822549	3.977830
C	-5.793593	4.869676	4.598359
C	-5.116570	3.615789	4.312743
C	-3.741508	3.606319	4.051476
C	-2.990774	4.850232	4.070768
C	-1.993098	4.789869	3.018948
C	-1.678409	5.937487	2.286467
C	-2.357684	7.190099	2.577115
C	-3.322542	7.245277	3.586893
C	-4.556937	7.993223	3.379502
C	-5.634385	7.262734	4.015714
C	-5.072288	6.064887	4.617212
C	-3.643516	6.056359	4.350773

Dimer **1**-$C_{s}^{\cdot+}$

C	-3.945370	4.224548	-10.712773
C	-5.349321	4.098833	-10.364037
C	-5.519779	2.859257	-9.625307
C	-4.200968	2.220332	-9.503179
C	-3.243553	3.067266	-10.178582
C	-3.325686	5.476686	-10.706154
C	-4.087382	6.659553	-10.335842
C	-5.435646	6.540939	-9.992796
C	-6.084701	5.238174	-10.013284
C	-7.010561	5.178298	-8.896505
C	-7.163456	3.983076	-8.183081
C	-6.400532	2.804214	-8.551791
C	-6.046788	2.082900	-7.337487
C	-4.850529	1.195894	-7.256987
C	-3.849161	1.535946	-8.350387
C	-2.535590	1.714776	-7.793719
C	-1.579258	2.512365	-8.463753
C	-1.942188	3.201879	-9.680459
C	-1.299540	4.503603	-9.673852
C	-1.979427	5.620335	-10.170924
C	-1.913306	6.885776	-9.463827
C	-3.210077	7.527905	-9.562365
C	-3.720249	8.243691	-8.473392
C	-5.124751	8.116770	-8.115074
C	-5.966014	7.286050	-8.859634
C	-6.935446	6.439041	-8.183898
C	-7.016774	6.452652	-6.784304
C	-7.164588	5.208310	-6.057791
C	-7.234462	4.000149	-6.739200
C	-6.514376	2.813458	-6.242966
C	-5.748723	2.918172	-5.025456
C	-4.507208	2.292679	-4.886772
C	-3.987161	1.312821	-5.884692
C	-2.596450	1.707885	-6.357407
C	-1.770319	2.554910	-5.635420
C	-0.812458	3.401991	-6.309992
C	-0.700852	3.372632	-7.705095
C	-0.526703	4.610004	-8.444015
C	-0.464746	5.828814	-7.760544
C	-1.167824	6.988341	-8.277519
C	-1.696084	7.736274	-7.152764
C	-2.950370	8.349604	-7.248338
C	-3.875224	8.288864	-6.126822
C	-5.219671	8.141345	-6.663646
C	-6.145869	7.323602	-6.010840
C	-5.765320	6.624551	-4.793368
C	-6.384735	5.315332	-4.817476
C	-5.710654	4.209346	-4.315783
C	-4.384593	4.365247	-3.762019
C	-3.629451	3.185075	-4.142014
C	-2.286847	3.304293	-4.480390
C	-1.646100	4.608458	-4.471168
C	-0.730187	4.667100	-5.596321
C	-0.561886	5.857451	-6.308178
C	-1.318618	7.041166	-5.929737
C	-2.206054	6.985897	-4.853207
C	-3.510977	7.623964	-4.953174
C	-4.474450	6.777484	-4.268149
C	-3.770807	5.623804	-3.741460
C	-2.370242	5.749340	-4.102694
C	-4.154406	-3.948648	-9.057972
C	-5.452988	-3.321511	-9.149039
C	-6.469110	-4.345214	-8.956380

C	-5.787813	-5.611580	-8.746619
C	-4.354918	-5.365022	-8.810330
C	-3.102835	-3.267101	-8.427772
C	-3.306313	-1.937723	-7.890066
C	-4.542604	-1.329731	-8.000398
C	-5.635751	-2.043314	-8.603845
C	-6.845747	-1.735609	-7.860465
C	-7.830787	-2.717827	-7.689345
C	-7.633109	-4.049513	-8.241983
C	-8.164926	-5.008275	-7.288190
C	-7.510242	-6.227732	-7.084600
C	-6.297937	-6.535186	-7.828031
C	-5.397092	-7.250612	-6.940418
C	-4.019183	-7.014816	-7.004003
C	-3.490081	-6.054146	-7.952426
C	-2.388569	-5.352224	-7.310833
C	-2.205998	-3.985987	-7.539387
C	-1.845650	-3.102729	-6.443555
C	-2.515429	-1.834271	-6.641683
C	-2.991616	-1.126151	-5.553968
C	-4.220839	-0.237649	-5.622165
C	-5.073963	-0.350517	-6.969741
C	-6.503879	-0.830951	-6.794672
C	-7.106150	-0.973571	-5.559346
C	-8.111331	-1.996646	-5.356277
C	-8.479384	-2.851486	-6.405387
C	-8.689012	-4.265470	-6.151764
C	-8.537827	-4.773902	-4.856681
C	-7.863206	-6.040070	-4.646868
C	-7.356680	-6.753638	-5.738973
C	-6.051629	-7.387301	-5.649614
C	-5.304478	-7.290162	-4.471011
C	-3.871843	-7.051900	-4.534905
C	-3.241012	-6.913316	-5.776346
C	-2.231617	-5.889953	-5.967461
C	-1.882260	-5.042293	-4.910138
C	-1.678854	-3.625452	-5.152721
C	-2.200914	-2.897248	-4.019245
C	-2.864408	-1.681258	-4.232998
C	-4.075718	-1.373947	-3.491486
C	-4.955997	-0.628977	-4.352424
C	-6.315660	-0.870352	-4.311841
C	-6.853666	-1.832678	-3.372495
C	-7.957498	-2.527212	-4.012371
C	-8.172513	-3.885501	-3.763851
C	-7.279902	-4.609796	-2.871940
C	-7.084950	-5.938777	-3.419203
C	-5.831597	-6.555130	-3.333018
C	-4.725507	-5.864520	-2.692579
C	-3.513111	-6.171752	-3.435959
C	-2.536073	-5.187444	-3.618217
C	-2.733520	-3.857801	-3.065301
C	-3.898151	-3.562358	-2.351948
C	-4.579365	-2.293899	-2.561513
C	-6.003657	-2.528558	-2.500500
C	-6.216834	-3.943033	-2.252435
C	-4.913825	-4.584551	-2.160320

Dimer **3**$^{\cdot+}$

C	2.684101	-1.523027	4.339012
C	3.084171	-0.258496	3.735763
C	2.551389	-0.193932	2.389057
C	1.796219	-1.447575	2.174926
C	1.889253	-2.253268	3.371660
C	2.359571	-1.587136	5.695584
C	2.414948	-0.383508	6.509623
C	2.805184	0.831241	5.934827
C	3.156551	0.894979	4.528038
C	2.667454	2.146547	3.998049
C	2.142520	2.187168	2.699113
C	2.136472	1.018410	1.845923
C	0.964639	1.111194	0.882894
C	0.174255	-0.228855	0.728838
C	0.608193	-1.420444	1.479863
C	-0.535228	-2.179688	1.891884
C	-0.441036	-2.994445	3.038489
C	0.792242	-3.021996	3.793265
C	0.452206	-3.082747	5.201808
C	1.219434	-2.377320	6.135228
C	0.567775	-1.653481	7.209685
C	1.306003	-0.422752	7.439848
C	0.621954	0.757846	7.770077
C	1.027563	2.019143	7.178812
C	2.097502	2.056807	6.277937
C	2.008519	2.865519	5.078705
C	0.853952	3.619055	4.828232
C	0.308168	3.664078	3.491895
C	0.932543	2.956744	2.448931
C	0.153743	2.250886	1.480434
C	-1.237182	2.207698	1.604276
C	-2.000781	1.014643	1.352577
C	-1.401928	-0.213457	0.751676
C	-1.741257	-1.457040	1.571105
C	-2.782988	-1.478711	2.491500
C	-2.690995	-2.278576	3.698532
C	-1.547174	-3.040408	3.965468
C	-0.999830	-3.092345	5.310997
C	-1.624604	-2.387688	6.343624
C	-0.829302	-1.657279	7.310817
C	-1.537048	-0.431240	7.647813
C	-0.823457	0.751072	7.876335
C	-1.318515	2.013211	7.347428
C	-0.173335	2.798368	6.916826
C	-0.257852	3.587940	5.767881
C	-1.493066	3.619914	5.002151
C	-1.140631	3.664369	3.595517
C	-1.898390	2.956537	2.663814
C	-3.045646	2.185571	3.090301
C	-3.092865	0.978904	2.271122
C	-3.492135	-0.241462	2.844634
C	-3.839334	-0.296348	4.243609
C	-3.344916	-1.554567	4.778604
C	-2.823320	-1.608084	6.071425
C	-2.768406	-0.401100	6.884346
C	-3.246335	0.811431	6.372592
C	-2.506048	2.045088	6.612536
C	-2.595504	2.862510	5.417153
C	-3.387313	2.135120	4.439186
C	-3.786371	0.865915	5.033405
C	2.193161	-1.949014	-4.026674
C	2.827787	-0.918157	-3.235149
C	3.407499	0.055495	-4.129999
C	3.127339	-0.367744	-5.491678
C	2.375463	-1.613133	-5.425648
C	0.983292	-2.517675	-3.596588
C	2.214524	-0.480891	-2.043430
C	2.226097	0.905768	-1.647476
C	2.751640	1.836446	-2.536474
C	3.351419	1.411964	-3.787719
C	3.033526	2.401515	-4.806506
C	2.769573	1.996539	-6.115177
C	2.812385	0.584145	-6.465186
C	1.746599	0.329026	-7.414054
C	1.027678	-0.871716	-7.354929
C	1.346286	-1.858309	-6.341078
C	0.096308	-2.454206	-5.893362
C	-0.082489	-2.772291	-4.545487
C	-1.352477	-2.498872	-3.886166
C	0.812680	3.350473	-2.172662
C	2.045729	3.097629	-2.800292
C	2.226787	3.441756	-4.189219
C	1.178313	4.033958	-4.915638
C	0.904581	3.615587	-6.271125
C	1.681322	2.611524	-6.862066
C	1.049638	1.581881	-7.662562
C	-0.337933	1.588601	-7.846152
C	-1.084239	0.348146	-7.783225
C	-0.414355	-0.861021	-7.538911
C	-0.989395	-1.841520	-6.641720
C	-2.213084	-1.583843	-6.012968
C	-2.403645	-1.927551	-4.615945
C	-3.207628	-0.894986	-4.004141
C	-2.930296	-0.493906	-2.690172
C	-2.959277	0.924248	-2.362574
C	-1.509054	3.361008	-2.492716
C	-0.271505	3.971923	-2.926016
C	-0.095400	4.311372	-4.264950
C	-1.157604	4.058725	-5.224474
C	-0.541119	3.626591	-6.464968
C	-1.147818	2.631884	-7.235235
C	-2.396539	2.029870	-6.796606
C	-2.354837	0.615403	-7.136277
C	-2.909645	-0.330457	-6.267386
C	-3.518727	0.096416	-5.023553
C	-3.562650	1.458833	-4.698001
C	-3.277342	1.877727	-3.346034
C	-2.535430	3.125045	-3.406050
C	-2.360899	3.480032	-4.801729
C	-2.993359	2.445865	-5.604467
C	-1.344547	-0.133019	-0.902067
C	0.214842	-0.151103	-0.796820
C	1.012271	1.208614	-0.771117
C	0.249086	2.383667	-1.286315
C	-1.922795	1.177761	-1.411871
C	-1.174246	2.354231	-1.495471
C	-1.090692	-2.081673	-2.522923
C	-1.898594	-1.133294	-1.902645
C	0.378713	-2.088326	-2.355361
C	0.988409	-1.093590	-1.624731

Dimer **4**$^{\cdot+}$

C	2.698010	-1.530367	4.493841
C	3.097423	-0.346146	3.752747
C	2.546081	-0.438133	2.421339
C	1.792156	-1.697868	2.334207

C	1.892154	-2.359484	3.613729
C	2.374107	-1.429415	5.847701
C	2.435465	-0.136974	6.511803
C	2.829620	1.002767	5.800276
C	3.163518	0.897664	4.394405
C	2.671493	2.086408	3.721412
C	2.121686	1.985627	2.425374
C	2.094928	0.708013	1.774275
C	0.978180	0.658383	0.810911
C	0.183148	-0.669337	0.718547
C	0.610745	-1.771162	1.602801
C	-0.538721	-2.456876	2.118599
C	-0.444999	-3.117210	3.362566
C	0.791698	-3.062753	4.121286
C	0.456461	-2.960461	5.526737
C	1.227682	-2.154083	6.372884
C	0.582484	-1.307989	7.360270
C	1.326664	-0.065047	7.445652
C	0.646192	1.146429	7.629491
C	1.051879	2.326457	6.884496
C	2.123895	2.261554	5.993316
C	2.033110	2.921672	4.701819
C	0.867588	3.621554	4.344875
C	0.334750	3.533132	3.001949
C	0.968437	2.765071	2.023939
C	0.192827	2.014311	0.936300
C	-1.217097	1.834345	1.374992
C	-1.923642	0.653580	1.294470
C	-1.387800	-0.624696	0.754581
C	-1.745839	-1.767236	1.711075
C	-2.779827	-1.667718	2.643481
C	-2.686221	-2.312769	3.935335
C	-1.540565	-3.045697	4.289901
C	-0.995609	-2.947581	5.634133
C	-1.614372	-2.125953	6.577092
C	-0.815454	-1.293978	7.460955
C	-1.515569	-0.040468	7.653941
C	-0.798823	1.156644	7.736540
C	-1.291684	2.348081	7.057068
C	-0.149676	3.068235	6.528098
C	-0.237393	3.706099	5.286086
C	-1.478029	3.664454	4.534279
C	-1.135664	3.554175	3.130998
C	-1.885350	2.720641	2.320271
C	-3.045574	2.023755	2.832529
C	-3.076493	0.731035	2.183471
C	-3.502609	-0.398809	2.858456
C	-3.847221	-0.291200	4.261644
C	-3.341321	-1.476663	4.928847
C	-2.814170	-1.380973	6.221079
C	-2.754650	-0.095162	6.888450
C	-3.234776	1.051501	6.245122
C	-2.487733	2.299572	6.331194
C	-2.587427	2.972388	5.051649
C	-3.395358	2.140678	4.177831
C	-3.795838	0.954099	4.912437
C	2.175509	-1.978792	-4.296115
C	2.813448	-1.054271	-3.389550
C	3.380057	0.030133	-4.156244
C	3.108440	-0.228957	-5.558631
C	2.360760	-1.476178	-5.645472
C	0.965710	-2.597381	-3.927129
C	2.195774	-0.769745	-2.161707
C	2.186620	0.569969	-1.640196
C	2.730201	1.615329	-2.366553
C	3.328159	1.343717	-3.652956
C	3.002828	2.443523	-4.537947
C	2.746314	2.196431	-5.890133
C	2.792707	0.833908	-6.407623
C	1.730888	0.691840	-7.386742
C	1.012162	-0.507449	-7.470271
C	1.329349	-1.606984	-6.577746
C	0.078171	-2.253678	-6.200068
C	-0.100601	-2.732725	-4.898409
C	-1.368010	-2.545350	-4.209623
C	0.824268	3.133081	-1.751100
C	2.017347	2.908483	-2.441994
C	2.195903	3.399555	-3.795904
C	1.164351	4.084372	-4.453183
C	0.888960	3.820044	-5.852357
C	1.663983	2.891419	-6.558691
C	1.034526	1.962998	-7.482780
C	-0.351754	1.989258	-7.663933
C	-1.096389	0.746937	-7.750177
C	-0.428821	-0.477761	-7.652695
C	-1.003469	-1.557789	-6.868279
C	-2.229685	-1.382042	-6.214343
C	-2.417625	-1.890630	-4.868948
C	-3.221126	-0.938059	-4.137372
C	-2.936597	-0.697503	-2.785206
C	-2.938728	0.674773	-2.314162
C	-1.493567	3.085510	-2.146331
C	-0.257660	3.770987	-2.473980
C	-0.097800	4.271442	-3.774586
C	-1.163679	4.124181	-4.758039
C	-0.553350	3.847497	-6.041919
C	-1.161317	2.948751	-6.929111
C	-2.409698	2.299022	-6.569788
C	-2.367356	0.935922	-7.068885
C	-2.924160	-0.107931	-6.316840
C	-3.535112	0.168122	-5.033257
C	-3.572190	1.482080	-4.552914
C	-3.278587	1.739595	-3.155190
C	-2.538622	2.973916	-3.069359
C	-2.365358	3.494544	-4.412833
C	-3.004677	2.569329	-5.333772
C	-1.341039	-0.539890	-0.954582
C	0.212611	-0.588963	-0.846210
C	1.018057	0.754787	-0.753344
C	0.246394	2.108492	-0.770012
C	-1.874520	0.814022	-1.368224
C	-1.170801	1.988646	-1.286445
C	-1.104081	-2.298419	-2.804282
C	-1.908018	-1.424929	-2.068710
C	0.365278	-2.330431	-2.640670
C	0.982252	-1.439665	-1.779824

Dimer **HPR-*C*$_s$**$^{\cdot+}$

C	2.628146	-1.588295	4.777243
C	3.131193	-0.456170	4.018442
C	2.724043	-0.613385	2.638712
C	1.949090	-1.871470	2.543159
C	1.899621	-2.454713	3.866626
C	2.181293	-1.412541	6.089612
C	2.209211	-0.089196	6.694259
C	2.697870	1.001415	5.967890
C	3.172135	0.815241	4.609157
C	2.775312	1.969792	3.835943
C	2.351319	1.800943	2.504422
C	2.378019	0.498272	1.884058
C	0.854664	-1.973700	1.696443
C	-0.358244	-2.595715	2.170422
C	-0.407156	-3.193490	3.443742
C	0.747045	-3.119630	4.310059
C	0.275323	-2.929060	5.669135
C	0.975648	-2.090410	6.542121
C	0.255126	-1.181301	7.414762
C	1.015998	0.053093	7.508523
C	0.349249	1.284635	7.564667
C	0.852960	2.416693	6.808652
C	2.004394	2.282213	6.026314
C	2.056909	2.875728	4.702951
C	0.949090	3.574691	4.201882
C	0.542239	3.417058	2.822188
C	1.253079	2.575527	1.978672
C	-1.508046	-1.904686	1.638408
C	-2.634711	-1.737303	2.430688
C	-2.684875	-2.320712	3.754141
C	-1.596104	-3.050894	4.252640
C	-1.181613	-2.886425	5.633378
C	-1.873953	-2.007097	6.472121
C	-1.145417	-1.140259	7.380413
C	-1.836715	0.136388	7.438864
C	-1.102883	1.327163	7.529085
C	-1.501460	2.485677	6.750690
C	-0.291699	3.155772	6.297523
C	-0.243833	3.717257	5.018797
C	-1.401454	3.643912	4.144359
C	-0.937518	3.460599	2.785819
C	-1.654191	2.660668	1.907325
C	-2.820021	1.952060	2.377399
C	-2.891947	0.652125	1.755026
C	-3.339110	-0.436335	2.489991
C	-3.803558	-0.253491	3.848201
C	-3.405833	-1.412132	4.629082
C	-3.014090	-1.260578	5.961944
C	-2.994413	0.062948	6.566434
C	-3.382123	1.179211	5.818478
C	-2.618948	2.417768	5.912613
C	-2.571372	3.011240	4.589236
C	-3.297898	2.147350	3.686830
C	-3.799193	1.019067	4.437949
C	2.144854	-1.958613	-4.604914
C	2.871140	-1.094410	-3.702575
C	3.372234	0.033822	-4.453862
C	2.955295	-0.126751	-5.834410
C	2.192544	-1.365504	-5.928420
C	0.975015	-2.591495	-4.160098
C	2.392950	-0.898972	-2.393336
C	2.464622	0.401115	-1.771205
C	2.911686	1.489539	-2.506224
C	3.376215	1.306531	-3.864454
C	2.978297	2.464828	-4.645703
C	2.586811	2.313004	-5.978565
C	2.567442	0.989274	-6.582690
C	1.409901	0.915326	-7.455307
C	0.676413	-0.275647	-7.545399
C	1.075074	-1.433855	-6.766557
C	-0.134519	-2.104364	-6.313596
C	-0.182530	-2.665484	-5.034703
C	-1.375453	-2.522868	-4.218068
C	1.079924	2.957582	-1.655475
C	2.206707	2.790190	-2.447548
C	2.256823	3.373424	-3.770991
C	1.167997	4.103256	-4.269927
C	0.753692	3.938383	-5.650717
C	1.446388	3.059024	-6.489093
C	0.718231	2.191832	-7.397327
C	-0.682317	2.232436	-7.431981

C	-1.442807	0.997823	-7.525599
C	-0.775756	-0.233546	-7.581304
C	-1.279252	-1.365504	-6.824982
C	-2.430856	-1.231172	-6.042868
C	-2.483458	-1.824264	-4.719412
C	-3.202137	-0.918242	-3.852722
C	-2.778449	-0.748882	-2.521152
C	-2.805496	0.553893	-1.901080
C	-1.282771	3.026357	-1.713754
C	-0.069896	3.648271	-2.187745
C	-0.021118	4.245719	-3.461277
C	-1.175061	4.171329	-4.327811
C	-0.703222	3.980717	-5.686844
C	-1.403185	3.141596	-6.559730
C	-2.608589	2.463516	-6.107178
C	-2.636105	1.140029	-6.711485
C	-3.124641	0.049498	-5.984885
C	-3.599281	0.235982	-4.626256
C	-3.558684	1.507461	-4.035848
C	-3.151637	1.665247	-2.656138
C	-2.376948	2.923490	-2.560794
C	-2.327602	3.506456	-3.884438
C	-3.055685	2.639593	-4.794959
C	-0.969012	-2.364770	-2.838307
C	-1.680149	-1.523110	-1.995115
C	0.510774	-2.407997	-2.801656
C	1.227056	-1.607598	-1.923279
C	1.333260	0.427969	0.822107
C	0.516591	-0.896709	0.721844
C	0.582136	1.814731	0.885193
C	-0.976172	1.860428	0.846936
C	-1.802439	0.519627	0.745264
C	-1.060486	-0.850613	0.683092
C	-1.009413	-0.761998	-0.901809
C	0.548632	-0.807185	-0.863504
C	1.374788	0.533758	-0.761942
C	0.632717	1.903720	-0.699627
C	-1.760995	0.624738	-0.838860
C	-0.944858	1.949667	-0.738498

Dimer **HPR-$D_{2h}^{\cdot+}$**

C	-4.837045	4.573415	-10.929337
C	-6.118119	4.531753	-10.245522
C	-6.266060	3.212513	-9.651639
C	-5.076576	2.439525	-9.972561
C	-4.194327	3.283362	-10.760685
C	-4.071273	5.745267	-10.906082
C	-4.560457	6.915868	-10.197843
C	-5.799256	6.881179	-9.549438
C	-6.596108	5.661762	-9.573969
C	-7.227493	5.519015	-8.277071
C	-7.355869	4.248100	-7.693062
C	-6.868291	3.076267	-8.399384
C	-6.314920	2.157542	-7.420198
C	-5.176273	1.399506	-7.739036
C	-4.546459	1.545691	-9.036551
C	-3.104479	1.469693	-8.842810
C	-2.257190	2.291368	-9.593278
C	-2.808691	3.210139	-10.574762
C	-2.015166	4.424763	-10.550609
C	-2.635088	5.669481	-10.713401
C	-2.231625	6.793098	-9.884911
C	-3.422936	7.563407	-9.564427
C	-3.563135	8.134399	-8.297977
C	-4.844420	8.090871	-7.615912
C	-5.946029	7.480182	-8.237372
C	-6.827659	6.640807	-7.459212
C	-6.565586	6.442115	-6.091446
C	-6.757715	5.145480	-5.488073
C	-7.115699	4.062045	-6.280577
C	-6.460598	2.746792	-6.109044
C	-5.470936	2.563559	-5.151958
C	-4.271284	1.837025	-5.490629
C	-4.133479	1.231830	-6.752877
C	-2.857036	1.277456	-7.427530
C	-1.770073	1.918052	-6.810058
C	-0.886483	2.761188	-7.595968
C	-1.120603	2.941554	-8.960580
C	-0.970066	4.260878	-9.554368
C	-0.572207	5.344682	-8.765043
C	-1.217388	6.639875	-8.934224
C	-1.360204	7.238751	-7.621821
C	-2.517391	7.968398	-7.303496
C	-3.139474	7.828694	-6.006933
C	-4.603658	7.905936	-6.203420
C	-5.466723	7.121247	-5.448778
C	-4.984571	6.310589	-4.295450
C	-5.842797	4.996809	-4.322187
C	-5.151983	3.610921	-4.142012
C	-3.598495	3.528861	-3.933885
C	-3.134972	2.440398	-4.838332
C	-1.914615	2.507113	-5.498617
C	-1.105593	3.745389	-5.473613
C	-0.484090	3.886075	-6.770104

C	-0.322766	5.155405	-7.349640
C	-0.806290	6.323776	-6.650689
C	-1.438459	6.171832	-5.403328
C	-2.591830	6.969443	-5.062874
C	-3.441365	6.228989	-4.088998
C	-2.746313	4.833494	-3.907024
C	-1.546772	4.871029	-4.789366
C	-3.804966	3.631392	-2.353898
C	-5.358332	3.713128	-2.562333
C	-5.942435	2.796818	-1.543311
C	-4.881757	2.139388	-0.819474
C	-3.606540	2.674400	-1.230510
C	-2.952408	4.936144	-2.327560
C	-3.647424	6.331672	-2.508214
C	-5.190626	6.412891	-2.714839
C	-6.048826	5.099179	-2.741620
C	-7.228481	5.379513	-1.876171
C	-7.795830	4.399398	-1.071344
C	-7.140812	3.083782	-0.901655
C	-7.344619	2.668506	0.467006
C	-6.337955	1.976026	1.159939
C	-5.081176	1.701726	0.502370
C	-4.018206	1.853698	1.469204
C	-2.799335	2.429219	1.073466
C	-2.594292	2.844436	-0.294769
C	-1.785108	4.082897	-0.269317
C	-2.017363	5.105308	-1.180595
C	-2.048452	6.475254	-0.729501
C	-3.062819	7.203919	-1.451888
C	-3.819504	8.166883	-0.796228
C	-5.283817	8.244137	-0.991725
C	-5.937687	7.355166	-1.835533
C	-7.175199	6.744886	-1.413674
C	-7.775329	7.111604	-0.195384
C	-8.389889	6.096135	0.628294
C	-8.386112	4.759375	0.197367
C	-8.115182	3.694462	1.147343
C	-7.850679	3.998717	2.484096
C	-6.795538	3.292781	3.193719
C	-6.058200	2.296485	2.545717
C	-4.616066	2.220384	2.738032
C	-3.975375	3.144597	3.569583
C	-2.704424	3.728275	3.170441
C	-2.132122	3.379775	1.945520
C	-1.513454	4.397467	1.114280
C	-1.483884	5.732589	1.549543
C	-1.753004	6.795054	0.608062
C	-2.522107	7.816274	1.280970
C	-3.547237	8.480483	0.587537
C	-4.809035	8.754175	1.252476
C	-5.874790	8.603243	0.277068
C	-7.108313	8.057535	0.669146
C	-7.311751	7.632619	2.039849
C	-8.108770	6.413008	2.014636
C	-7.837470	5.385117	2.923379
C	-6.772817	5.534593	3.900918
C	-6.130011	4.244551	4.067265
C	-4.744190	4.171411	4.251992
C	-3.950543	5.386005	4.277078
C	-2.688618	5.114205	3.610195
C	-2.083921	6.095813	2.818214
C	-2.729259	7.391403	2.651215
C	-3.950247	7.647267	3.283963
C	-5.006703	8.350922	2.574607
C	-6.279212	7.769808	2.973087
C	-6.007060	6.706569	3.925159
C	-4.570766	6.630722	4.116612

C_{120}-NT-$D_{5d}^{\cdot+}$

C	12.945054	12.664911	19.067770
C	13.623131	12.396881	17.863340
C	12.930910	12.642120	16.626456
C	13.624409	12.432636	15.391682
C	12.938899	12.631955	14.164314
C	13.625599	12.401045	12.929561
C	12.908394	12.565797	11.693399
C	13.614313	12.392803	10.487720
C	13.157332	12.964673	9.245608
C	14.314981	13.351099	8.462043
C	14.290850	14.531052	7.707673
C	15.450295	15.408628	7.699747
C	14.974440	16.782839	7.689974
C	15.653340	17.760457	8.428084
C	14.909228	18.736631	9.200559
C	15.611971	18.975999	10.436210
C	14.934031	19.244250	11.640663
C	15.626436	18.999300	12.877555
C	14.932987	19.208920	14.112422
C	15.618378	19.009074	15.339788
C	14.931826	19.240216	16.574610
C	15.648986	19.075007	17.810761
C	14.943059	19.247895	19.016443
C	15.399924	18.675873	20.258537
C	14.242214	18.289468	21.041984
C	14.266229	17.109528	21.796328
C	13.106671	16.232063	21.804371
C	13.582438	14.857838	21.814092
C	12.903510	13.880223	21.076029
C	13.647560	12.903933	20.303590
C	11.760295	13.492436	19.063095

C	11.734223	14.238333	20.296481
C	11.278279	15.556004	20.287126
C	11.971812	16.570727	21.057172
C	11.956179	17.788670	20.269868
C	13.067628	18.630623	20.262602
C	13.498008	19.219477	19.018863
C	12.795422	19.018714	17.815617
C	13.501275	19.211913	16.577069
C	12.820110	18.953432	15.344768
C	13.492661	19.180201	14.115005
C	12.803574	18.942623	12.882868
C	13.480784	19.215053	11.643456
C	12.808898	18.920913	10.441659
C	13.515312	18.709543	9.203154
C	12.806719	17.705227	8.433192
C	13.520344	16.754597	7.692675
C	13.098266	15.363097	7.704033
C	11.980047	14.980594	8.455176
C	12.013982	13.762733	9.242223
C	11.316287	13.998918	10.481182
C	11.717365	13.398941	11.689801
C	11.312235	14.020605	12.922047
C	11.758796	13.458267	14.160311
C	11.331261	14.038772	15.383358
C	11.758597	13.462638	16.622105
C	11.283603	14.033220	17.854161
C	10.809637	15.407006	17.844339
C	10.844375	16.142496	19.043952
C	11.264126	17.525218	19.033257
C	11.637987	18.139677	17.823226
C	11.253117	17.502379	16.592607
C	11.673458	18.081333	15.352919
C	11.263991	17.484768	14.131545
C	11.664299	18.077005	12.891514
C	11.206870	17.488707	11.661159
C	11.657338	18.047924	10.450689
C	11.655253	17.301878	9.217149
C	11.250623	15.967534	9.227922
C	10.843901	15.364520	10.472452
C	10.785650	16.097813	11.672414
C	10.845869	15.373119	12.913176
C	10.846731	16.106095	14.142443
C	10.861894	15.400468	15.374021
C	10.838439	16.133146	16.603285
C	15.059376	12.421138	10.485196
C	15.489557	13.010079	9.241427
C	16.601070	13.851964	9.234042
C	16.585277	15.070029	8.446832
C	17.278658	16.084719	9.217016
C	16.822669	17.402443	9.207606
C	16.796425	18.148163	10.441082
C	17.272738	17.607173	11.650034
C	16.798379	18.178322	12.882085
C	17.225046	17.601596	14.120728
C	16.798137	18.182411	15.343760
C	17.244342	17.619575	16.581946
C	16.839742	18.241459	17.814245
C	17.240733	17.641399	19.022851
C	16.543126	17.877614	20.261895
C	16.576982	16.659858	21.049071
C	15.458797	16.277472	21.800257
C	15.036483	14.885964	21.811492
C	15.750113	13.935237	21.071019
C	15.041480	12.931041	20.301010
C	15.748060	12.719452	19.062592
C	15.076394	12.425567	17.860626
C	15.754209	12.697256	16.621283
C	15.064881	12.460987	15.389041
C	15.737830	12.686783	14.159272
C	15.056257	12.429244	12.926967
C	15.762159	12.621822	11.688411
C	16.919670	13.500674	11.680678
C	17.293224	14.115369	10.470650
C	17.712635	15.498134	10.460121
C	17.747037	16.233407	11.659901
C	17.718851	15.507082	12.900847
C	17.694372	16.239615	14.130113
C	17.710340	15.533951	15.361628
C	17.710778	16.267032	16.590875
C	17.771221	15.542446	17.831653
C	17.713162	16.275785	19.031643
C	17.306286	15.672837	20.276174
C	16.901568	14.338489	20.287095
C	16.899598	13.592336	19.053595
C	17.350028	14.151548	17.843093
C	16.893474	13.562664	16.612665
C	17.293455	14.155197	15.372643
C	16.884577	13.558354	14.151077
C	17.304590	14.137743	12.911401
